# Genome-wide analysis in chicken reveals that local levels of genetic diversity are mainly governed by the rate of recombination

**DOI:** 10.1186/1471-2164-14-86

**Published:** 2013-02-08

**Authors:** Carina F Mugal, Benoit Nabholz, Hans Ellegren

**Affiliations:** 1 Department of Evolutionary Biology, Evolutionary Biology Centre, Uppsala University, Norbyvagen 18D, SE-752 36, Uppsala, Sweden; 2 Present address: Institut des Sciences de l’Evolution, Université Montpellier II, Place Eugène Bataillon, 34095, Montpellier cedex 5, France

**Keywords:** Genetic diversity, Recombination rate, Chicken, Mutation, Selection, Gene density

## Abstract

**Background:**

Polymorphism is key to the evolutionary potential of populations. Understanding which factors shape levels of genetic diversity within genomes forms a central question in evolutionary genomics and is of importance for the possibility to infer episodes of adaptive evolution from signs of reduced diversity. There is an on-going debate on the relative role of mutation and selection in governing diversity levels. This question is also related to the role of recombination because recombination is expected to indirectly affect polymorphism via the efficacy of selection. Moreover, recombination might itself be mutagenic and thereby assert a direct effect on diversity levels.

**Results:**

We used whole-genome re-sequencing data from domestic chicken (broiler and layer breeds) and its wild ancestor (the red jungle fowl) to study the relationship between genetic diversity and several genomic parameters. We found that recombination rate had the largest effect on local levels of nucleotide diversity. The fact that divergence (a proxy for mutation rate) and recombination rate were negatively correlated argues against a mutagenic role of recombination. Furthermore, divergence had limited influence on polymorphism.

**Conclusions:**

Overall, our results are consistent with a selection model, in which regions within a short distance from loci under selection show reduced polymorphism levels. This conclusion lends further support from the observations of strong correlations between intergenic levels of diversity and diversity at synonymous as well as non-synonymous sites. Our results also demonstrate differences between the two domestic breeds and red jungle fowl, where the domestic breeds show a stronger relationship between intergenic diversity levels and diversity at synonymous and non-synonymous sites. This finding, together with overall lower diversity levels in domesticates compared to red jungle fowl, seem attributable to artificial selection during domestication.

## Background

Modern population genetics have attempted to explain patterns of genetic variation in light of evolutionary forces thought to affect DNA sequence evolution. One obvious factor to form a candidate for governing local polymorphism levels is the rate of mutation since, in the absence of selection, sequence divergence should be proportional to the mutation rate [1]. Another obvious factor is selection since both positive and negative selection reduces levels of genetic diversity at target loci. Selection should also affect diversity levels in regions linked to target loci. In the absence of recombination, the entire haplotype within which a selected allele is contained will be subject to change in frequency by selection. From this follows that recombination should itself be a factor of importance for levels of polymorphism. Specifically, when the local recombination rate is high, only regions within a relatively short physical distance from loci under selection are expected to show reduced polymorphism levels. There is well-developed theory for the expected effects of both types of selection relevant in this context, i.e. background selection arising from purifying selection [2] and selective sweeps arising from positive selection [3,4].

Empirically, one of the clearest patterns that have emerged from studies of the distribution of levels of polymorphism across the genome is the positive effect of recombination rate on genetic diversity. This relationship was first observed in *Drosophila melanogaster*[5,6] and then confirmed in various organisms including mouse [7], human [8-10], nematodes of the genus *Caenorabditis*[11,12], sea beet [13] and grasses [14,15]. However, a direct effect of recombination on mutation rate, i.e., a neutral scenario, has also been proposed to explain the correlation between recombination and polymorphism [9,16], although this possible mutagenic effect of recombination is debated [17-19]. A recent large-scale analysis failed to demonstrate a relationship between recombination hotspots and mutation rate in the human genome [20]. However, the fact that recombination rate as well as the rate of substitution often covary with several other genomic features impedes the understanding of any causal relationships [9,21].

The possibility to capture patterns of sequence polymorphism across whole genomes allows critical tests of the importance of different evolutionary factors in shaping diversity levels [22]. This information is especially important when making inferences of selection, not least when it comes to detecting signs of positive selection in searches for candidate loci for adaptive evolution. It is then imperative that variation in polymorphism caused by different factors can be distinguished from each other. Here we analyze genome-wide patterns of genetic diversity in domestic chicken *G. gallus domesticus* and its wild ancestor the red jungle fowl *G. gallus gallus*. This system is of particular interest given that intense artificial selection during domestication may have left strong footprints on patterns of genetic diversity within the genome [23-26]. We examine how nucleotide diversity in chicken is related to recombination rate, divergence in intergenic regions and at synonymous and non-synonymous sites, gene density and local GC content.

## Results

Using published information on a total of 7.2 million chicken SNPs obtained from re-sequencing of pooled samples [25], we derived genome-wide estimates of local (1 Mb windows) diversity level for three chicken populations (red jungle fowl [RJF], broiler and layer) based on non-repetitive and putatively non-functional sites in the genome (see Materials and Methods). Basically, this represents diversity in intergenic regions, which in the following we simply refer to as ‘diversity’. We also estimated diversity at synonymous and non-synonymous sites, which we refer to as p_S_ and p_N_, respectively. Domestic breeds had lower diversity levels than RJF (electronic Additional file 1: Table S1), however, principal component analysis (PCA) showed that 88.8% of the variation was common to all three populations, represented by principal component (PC) I (see biplots in the Additional file 1: Figure S1). Pairwise correlations between local diversity level and p_S_ and p_N_, respectively, had correlation coefficients in the range 0.118-0.454 and generally showed a stronger relationship in the domestic breeds than in RJF. For all three populations the strongest correlation was found between diversity level and p_S_ (Table 1).

**Table 1:** **Pairwise Pearson correlation coefficients (and associated *p*-values) between local diversity level and p**_**S **_**and p**_**N**_**, based on 1 Mb windows for three chicken populations**

	**Red jungle fowl**	**Broiler**	**Layer**
diversity level – p_S_	0.237 (1.17 · 10^-12^)	0.454 (< 2.2 · 10^-16^)	0.403 (< 2.2 · 10^-16^)
diversity level – p_N_	0.137 (4.68 · 10^-05^)	0.213 (1.65 · 10^-10^)	0.184 (3.98 · 10^-08^)
p_S_ – p_N_	0.118 (4.36 · 10^-04^)	0.199 (2.70 · 10^-09^)	0.201 (1.67 · 10^-09^)

In order to investigate the causes of local variation in diversity level we performed multi-linear regression analysis using a number of candidate explanatory variables selected to represent the effects of variation in mutation rate (intergenic divergence and the synonymous substitution rate, d_S_), local DNA composition (GC content), recombination and selection (gene density and the non-synonymous substitution rate, d_N_), respectively. Here, intergenic divergence, d_S_ and d_N_ represent chicken-specific divergence estimates based on triple-alignments between chicken, turkey and zebra finch. Gene density represents the proportion of genic sites in the chicken sequence and GC content is computed based on non-genic sites in the chicken sequence. Then, using diversity levels as response variable, regression analysis was performed for each population separately as well as for PC I of diversity levels of all three populations. The latter was considered an appropriate representative of the genetic variation common to all populations. Recombination rate was found to be the main explanatory variable followed by gene density, both being positively correlated with diversity. GC content showed a statistically significant impact in RJF and for common genetic variation, whereas divergence showed a significant and unexpected negative impact in layer and for common genetic variation. d_S_ and d_N_ were not significantly correlated with diversity level at a *p*-value threshold of 0.001 in any population (Table 2). Overall, similar patterns were found for regression analysis based on 500 kb and 250 kb windows. However, the amount of genetic variation explained, represented by multiple *R*^*2*^, decreased from 15% for analyses based on 1 Mb windows to 8% and 5% for 500 kb and 250 kb windows, respectively. The decrease for the smaller window sizes is likely a result of a reduction in the signal-to-noise ratio, which motivated further analyses to be focused on 1 Mb windows (for results based on 500 kb and 250 kb windows, see Additional file 1: Tables S2 and Additional file 1: Table S3).

**Table 2:** **Estimates, by which we refer to multiple regression coefficients, and *p*-values in multi-linear regression analysis for six possible explanatory variables of chicken diversity levels in 1 Mb windows**

	**Red jungle fowl**		**Broiler**		**Layer**		**Common**	
	**Estimate**	***p*-value**	**Estimate**	***p*-value**	**Estimate**	***p*-value**	**Estimate**	***p*-value**
recombination rate	**3.29 · 10**^**-05**^	**2.51 · 10**^**-16**^	**3.07 · 10**^**-05**^	**6.07 · 10**^**-16**^	**2.32 · 10**^**-05**^	**8.40 · 10**^**-10**^	**5.03 · 10**^**-05**^	**9.45 · 10**^**-16**^
divergence	−7.94 · 10^-06^	1.68 · 10^-02^	−7.77 · 10^-06^	1.33 · 10^-02^	**−1.82 · 10**^**-05**^	**1.07 · 10**^**-08**^	**−1.93 · 10**^**-05**^	**2.09 · 10**^**-04**^[27]
gene density	**2.30 · 10**^**-05**^	**4.45 · 10**^**-08**^	**2.11 · 10**^**-05**^	**1.07 · 10**^**-07**^	**2.19 · 10**^**-05**^	**3.95 · 10**^**-08**^	**3.81 · 10**^**-05**^	**6.54 · 10**^**-09**^
GC content	**−1.68 · 10**^**-05**^	**7.10 · 10**^**-04**^	−1.50 · 10^-05^	1.37 · 10^-03^	−1.31 · 10^-05^	5.49 · 10^-03^	**−2.60 · 10**^**-05**^	**7.94 · 10**^**-04**^
d_S_	−7.23 · 10^-06^	8.18 · 10^-02^	−7.18 · 10^-06^	6.76 · 10^-02^	−1.01 · 10^-05^	1.03 · 10^-02^	−1.41 · 10^-05^	3.00 · 10^-02^
d_N_	−3.66 · 10^-06^	3.70 · 10^-01^	−4.64 · 10^-06^	2.29 · 10^-01^	−4.08 · 10^-06^	2.92 · 10^-01^	−7.13 · 10^-06^	2.63 · 10^-01^
	Multiple *R*^*2*^ = 0.1513		Multiple *R*^*2*^ = 0.1509		Multiple *R*^*2*^ = 0.1601		Multiple *R*^*2*^ = 0.1693								
	*p* < 2.2 · 10^-16^		*p* < 2.2 · 10^-16^		*p* < 2.2 · 10^-16^		*p* < 2.2 · 10^-16^								

Common variation reflects PC I of diversity level of all three populations. Estimates and *p*-values significant at a threshold < 0.001 are highlighted in bold.

As multi-linear regression analysis is sensitive to multi-collinearity in the explanatory variables, we performed partial least square regression (PLSR) analysis, a regression setup that accounts for multi-collinearity in the explanatory variables and allows dissection of the interrelationships between explanatory variables. PLSR groups together explanatory variables into PCs based on their correlations with each other. Subsequent regression analysis and the number of significant PCs then illustrate the number of independent effects on the response variable. Each significant PC represents an independent effect by one of the contributors to the respective PC on the response variable, most likely the main contributor, which we refer to as the true explanatory variable. The remaining contributors to the PC are likely to be dragged by the true explanatory variable via their correlations to the true explanatory variable. As such PLSR enables us to quantify a lower bound of the amount of variation explained by the true explanatory variable, where the upper bound is given by the *R*^*2*^ obtained by simple linear regression.

**Figure 1:**
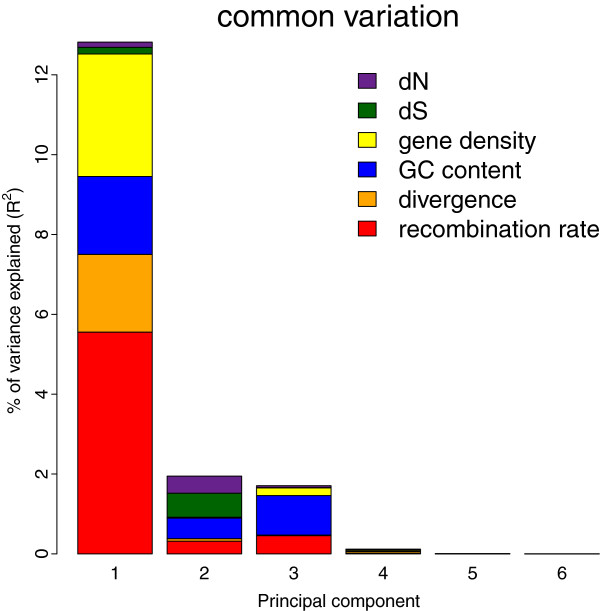
Amount of common genetic variation explained by the different explanatory variables based on PLSR analysis.

In agreement with the results of the multi-linear regression analysis, the PLSR analysis showed that recombination rate explained most of the variation in diversity level (6%) (Table 3, and visualized in Figure 1 and Additional file 1: Figure S2). Gene density (3%), GC content (3%) and divergence (2%) explained part of the variance, whereas the impact of d_S_ and d_N_ on diversity was < 1% and considered negligible. Figure 1 illustrates that most of the explanatory variables grouped together in PC I, which accentuates the complexity associated with determining independent effects of correlated explanatory variables. Based on PC I and in agreement with the multi-linear regression analysis the relationships between recombination rate, GC content and gene density, and diversity was positive, while divergence, d_S_ and d_N_ showed a negative relationship to diversity. Since recombination rate constitutes the main contributor to PC I it is likely to drag other correlated variables with it, like gene density, GC content and divergence. As recombination rate and gene density were positively correlated (*r* = 0.40, *p* < 2.2e-16), this could explain why we find a positive correlation between gene density and diversity level, despite theory predicting the opposite. Similarly, this could lead to a negative correlation between divergence and diversity level, as recombination rate and divergence were negatively correlated (*r* = −0.28, *p* < 2.2e-16). Note that correlations between gene density and recombination rate, and between divergence and recombination rate, do not need to be causal. However, the negative correlation between recombination rate and divergence argues against a mutagenic effect of recombination (for point mutations) in birds. Also note that correlations might be caused indirectly via a third parameter, like GC content (which itself is strongly correlated with recombination rate [*r* = 0.61, *p* < 2.2e-16], gene density [*r* = 0.66, *p* < 2.2e-16] and divergence [*r* = −0.27, *p* < 2.2e-16]), and thereby blur any true effects of mutation rate and/or gene density on diversity level. Based on PC II and III we again found a positive relationship between recombination rate and gene density, and diversity, and a negative relationship between divergence, d_S_ and d_N,_ and diversity. However, opposite to the results based on PC I, GC content now showed a negative relationship to diversity. This suggests that after correction for the effect of recombination rate on diversity, GC content shows an independent negative effect on diversity.

**Table 3:** Percentage of genetic variation explained by six possible explanatory variables according to PLSR analysis of chicken diversity level in 1 Mb windows

	**Red jungle fowl**	**Broiler**	**Layer**	**Common variation**
recombination rate	6.30	6.16	4.48	6.34
divergence	(1.29)	(1.37)	3.62	2.06
gene density	3.02	2.91	2.93	3.31
GC content	3.12	(3.06)	(3.13)	3.48
d_S_	(0.69)	(0.65)	(0.86)	(0.78)
d_N_	(0.49)	(0.58)	(0.66)	(0.61)

Values in parentheses are for parameters that did not meet the *p* < 0.001 threshold in multi-linear regression.

**Figure 2:**
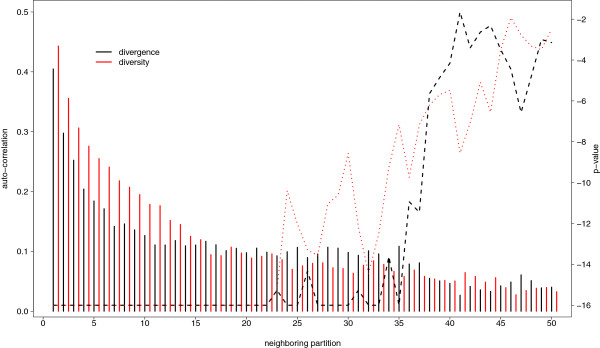
**Pearson correlation coefficients (bars) and their *p*-values (dashed lines) for divergence (black) and diversity level (red) of neighboring windows of size 100 kb.** The x-axis gives the number of the neighboring windows, starting from 1, i.e. nearest neighbor, up to 50, i.e. windows separated by 49 windows. The y-axis indicates the correlation coefficient on the left side and the related *p*-value (log-scale) on the right.

We tested if the lack of, or a negative, correlation between divergence and diversity level could arise as a result of differences in the range of spatial variation of the two parameters by performing an autocorrelation analysis based on 100 kb windows. However, the patterns were similar for both diversity and divergence (Figure 2). Correlations decreased from r > 0.4 between adjacent windows to r < 0.1 already at a distance of about 2 Mb, where *p*-values stayed low for a long distance.

Motivated by the results of the regression analysis, we investigated the impact of the explanatory variables for the possibility to identify candidate loci for selective sweeps. Based on the observation of locally reduced levels of genetic diversity, Rubin and colleagues identified 42 genomic regions as putative locations for selective sweeps in chicken [25]. Table 4 lists mean values for explanatory variables, and for p_S_ and p_N_, in these candidate regions together with genome-wide averages. This clearly highlights the importance of recombination rate for the ability to identify possible targets of positive selection: the mean recombination rate of candidate loci was reduced to 68% of the genome-wide average. Other explanatory variables also showed significant differences, but the effects were lower and are likely related to the correlation between explanatory variables.

**Table 4:** **Averages of diversity level, p**_**S**_**, p**_**N **_**and the six explanatory variables used for the regression analysis**

	**Genome-wide**	**Candidate loci**	***p***
diversity level × 10^4^	3.69/3.42/3.06	3.53/3.25/2.71	< 0.0001
p_S_ × 10^4^	5.12/5.11/4.66	5.30/5.00/4.50	0.338/0.395/0.167
p_N_ × 10^5^	5.50/5.38/5.01	4.00/4.87/4.46	0.005/0.006/0.006
recombination rate	1.154	0.781	< 0.0001
divergence	0.038	0.040	< 0.0001
gene density	0.026	0.022	< 0.0001
GC content	0.408	0.392	< 0.0001
d_S_ × 10^2^	4.87	4.60	< 0.0001
d_N_ × 10^2^	0.90	0.77	< 0.0001

The data are divided into genome-wide and candidate loci for selective sweeps identified by Rubin and colleagues [25]. *p*-values are based on bootstrapping. For diversity level, p_S_ and p_N_ means are reported for each of the three populations broiler, layer and RJF separately. Note that the absolute values of our diversity estimates are not directly comparable to previous estimates in chicken [28] since different sequencing methodologies and sampling design were used.

## Discussion

### Recombination as a determinant of levels of genetic diversity in chicken

Diversity levels are expected to reflect the product of the mutation rate and N_e_[29]. As a consequence, intra-genomic variation in the two latter parameters should lead to genomic heterogeneity in diversity levels. This heterogeneity can thus be framed either under a neutral scenario and reflect a mutation-driven pattern or under a model invoking natural selection causing variation in N_e_, or both. It is well established that there is significant variation in the rate of mutation across the genome (e.g., [30]), providing a basis for variation in diversity level. N_e_ is also expected to vary among genomic regions, in this case for reasons related to the incidence and efficiency of natural selection [29]. Importantly, with more functionally important sites follows more targets for selection. Moreover, when recombination rate is low, selection at linked sites will lower N_e_ over larger physical distances along chromosomes [29]. Whether mutation or selection is the main factor governing diversity levels is a matter of on-going debate [22].

We found that recombination rate had the strongest effect on diversity levels in two domestic chicken breeds and in RJF. In contrast, divergence, taken as a proxy for the rate of mutation, had either no effect or an unexpected minor negative effect. This supports a selection model where the effect of background selection and/or selective sweeps is (physically) more widely reaching in genomic regions with low recombination rates. A positive correlation between diversity and recombination has been frequently observed in previous work, however, that the effect of recombination is indirect via selection has been difficult to disentangle from a possible direct mutagenic effect of recombination [9,18,31]. In our case, the negative correlation between recombination rate and divergence strengthens the selection model and argues against a mutagenic effect. Moreover, this interpretation is supported by the positive correlation of both p_S_ and, in particular, p_N_ with intergenic diversity level, showing that selective events that reduce the diversity within coding regions also reduce diversity at nearby linked sites (cf. [32]), or vice versa.

As mentioned above, selective effects are expected to increase with gene density, each coding site representing a potential target for selection. In this respect, the positive correlation between gene density and diversity goes against the theoretical expectation [33]. We suggest that this is a statistical artifact caused by collinearity of explanatory variables, like the relatively strong positive correlation between recombination rate and gene density (r = 0.4). Estimates from multiple regressions should be interpreted cautiously if explanatory variables are correlated since they may lead to spurious and non-causative correlations, which very well might be the case here. Moreover, this might explain a similarly surprising result recently reported for Asian rice (*Oryza sativa*) where the association between gene density and recombination rate could potentially explain a negative relationship between recombination rate and polymorphism [21], similar to earlier findings in *Arabidopsis thaliana* and *A. lyrata*[34,35].

Several studies have shown that recombination rate is correlated with chromosome size [27,36,37], which is not unexpected given the requirement of at least one recombination event per chromosome (or chromosome arm) for successful meiosis; as a consequence, smaller chromosomes will have a higher recombination rate per physical distance compared to larger chromosomes. This is confirmed in our data, with a negative correlation between chromosome size and recombination rate (*r* = −0.31, *p* < 2.2e-16). As correlations are transitive relations, a correlation between chromosome size and recombination rate together with a correlation between recombination rate and diversity will lead to a correlation between chromosome size and diversity; this has been empirically demonstrated in previous analyses of birds [38]. In our main analysis we did not include chromosome size as candidate explanatory variable as we had no *a priori* reason to expect it to assert a direct effect on diversity level (in contrast to an indirect effect, via recombination). This was subsequently justified by a multi-linear regression analysis including chromosome size as explanatory variable (electronic Additional file 1: Table S4). This analysis suggested that in the RJF and broiler population there was no and in the layer population in fact an unexpected positive effect of chromosome size on diversity. Thus, we conclude that chromosome *per se* does not explain a negative correlation between chromosome size and diversity, as was also suggested by Megens et al. [39].

### The absence of an effect of divergence on diversity levels

We approximated local mutation rate by divergence estimates of the chicken branch after the split from turkey based on CpG-masked intergenic sequences as well as divergence at synonymous sites, d_S_. Using these estimates we failed to find a persuasive effect of divergence on level of genetic diversity. This is surprising considering theory (that diversity and divergence are correlated is a basic tenet of the neutral theory of molecular evolution), and empirical evidence from some [9,12,18,31] but not all previous studies [6,40]. The lack of a significant effect could be explained by several factors. First, in theory, it could reflect a lack of local variation in mutation rate across the chicken genome. However, this is clearly not in line with earlier observations from avian genomes [41,42]. Also, the auto-correlation analysis of divergence and diversity level (Figure 2) suggests that the two vary on a similar scale. Second, sequence features such as the local GC content appear to be strongly related to divergence in avian genomes. However, GC content is strongly correlated also with recombination rate via GC-biased gene conversion (gBGC), a process linked to recombination mimicking natural selection and leading to high GC content in high recombining regions [43]. As a consequence, the covariation of recombination rate, GC content and divergence together with a strong impact of recombination rate on diversity could blur independent signals between divergence and diversity as suggested by the PLSR analysis (Figure 1). Thus, taken together the positive correlation between recombination rate and diversity and the absence of correlation between divergence and diversity support a selection model, where the weaker impact of mutation rate on diversity, if any, becomes indistinct by the stronger impact of recombination rate on diversity.

### The impact of genomic features on identifying targets of adaptive evolution

There is considerable current interest in using population genomic data to identify regions that have been subject to recent events of positive selection. One means to do so is to search for outlier regions of nucleotide diversity, specifically regions of reduced diversity. The demonstration of recombination rate having a large impact on diversity level in the chicken genome should have at least two implications in this context. First, footprints of selection (selective sweeps) will be most easily seen in regions of low recombination, even if they occur less frequently in such regions due to Hill-Robertson interference. In addition, regions with low recombination and an associated reduced N_e_ are more likely to show hard sweeps than soft sweeps [44]. This should be manifested both in diversity level being reduced over a larger genomic region and the reduction being visible over a longer time scale since the sweep. Second, and as consequence of the former, studies of the genomic distribution of adaptively evolving loci will be biased towards regions with low rate of recombination. This was confirmed when we analyzed the location of candidate loci for selective sweeps identified by Rubin et al. [25], with recombination rate being significantly lower in these candidate regions compared to the genomic average. This emphasizes the necessity of a rigorous statistical framework that incorporates genomic features such as recombination rate when interpreting polymorphism levels.

### The footprint of domestication on patterns of chicken diversity

Although a significant part of the variation in diversity level was common to all three populations, we observed several important differences between domesticates and their wild ancestor. Rubin and colleagues found significantly lower overall heterozygosity in the two domestic breeds than in RJF [25]. Our results corroborate this observation, with the most pronounced difference seen in intergenic regions, with diversity level of the broiler and the layer being 83-93% of RJF. The difference is clearly in the expected direction given bottlenecks during domestication and genetic drift in closed commercial populations. Microsatellite-based genotyping has revealed this to be a common feature among chicken breeds [45] and, to a varying extent, the same trend has also been seen among other domestic animals and plants [46-48].

Strong artificial selection for traits of agronomical interest during domestication should also act to lower N_e_[25,49]. If artificial selection occurs frequently genome-wide it could create a stronger link between polymorphism at functional and neutral sites in domesticates than in natural populations [21]. In agreement with this prediction, the correlation of p_S_ and p_N_ to diversity level was stronger in the two domestic chicken breeds than in RJF (cf. Figure 2).

## Conclusions

Two previous studies have sought to address the influence of recombination on chicken diversity levels. Fang et al. [50] used low-coverage genome sequence data from three birds to obtain polymorphism estimates and made pairwise linear regression between diversity and recombination rate estimates from a medium-density linkage map. Rao et al. [51] sequenced 15 introns, and used data from Sundström et al. [52] for another 14 introns, and performed pairwise linear regression between diversity and recombination rate, and between diversity and chicken-turkey divergence. Similar to our findings, these two studies reported a correlation between diversity and recombination. However, our study adds to previous work in several ways. Notably, by the combined use of a genome-wide approach for diversity estimation from population samples and with the access to divergence data from across the genome, we were able to address and quantify the role of mutation and recombination on diversity level in a rigorous statistical framework. In addition we considered possible impacts of d_S_, d_N_, gene density and the local GC content. Based on our analysis we suggest that local levels of genetic diversity in the chicken genome are mainly governed by the rate of recombination. The fact that divergence and recombination rate were negatively correlated argues against a mutagenic role of recombination and for a selection model. In support of the selection model, divergence, taken as a proxy for the rate of mutation, had either no effect or an unexpected minor negative effect. Moreover, by including genome-wide estimates of p_S_ and p_N_ we were able to directly study the role of selection and to integrate information from functional sites in the genome. In addition, the genome-wide approach allowed us to test for possible effects of various genomic features on the ability to identify target loci of adaptive evolution. Further, we showed that artificial selection during domestication is likely to explain several differences in levels of diversity between domestic breeds and the wild ancestor (red jungle fowl), for example a stronger relationship between recombination rate and intergenic diversity, as well as a stronger relationship between intergenic diversity levels and diversity at synonymous as well as non-synonymous sites.

## Methods

### Short read sequences and read mapping

We used a dataset by Rubin and colleagues available at the European Nucleotide Archive (http://www.ebi.ac.uk/ena/) under the study accession number SRP001870 [25]. This dataset is composed of 35 bp reads obtained by SOLiD sequencing technology of genomic DNA pools of unrelated chicken from the red jungle fowl (RJF; 8 males and 1 female) and two domesticated populations, broiler (24 males and 18 females) and layer (29 males) (accession numbers SRR035386, SRR035383 and SRR035384 for RJF, SRR035377, SRR035378, SRR035387, SRR035381, SRR035382, SRR035379 and SRR035380 for broiler and SRR035375, SRR035376, SRR035389, SRR035390 and SRR035385 for layer).

The reads were mapped against the chicken reference genome (WUGSC 2.1, May 2006 version; [27]) downloaded at the UCSC Genome Browser website (http://genome.ucsc.edu/) [53]. The mapping was performed using the software BWA [46] allowing for a maximum of four mismatched bases and not allowing for insertions/deletions. Reads that mapped at several locations in the genome were excluded. Further, a genomic position had to fulfill four criteria in order to be included for downstream diversity level computation: (i) be covered by more than 4 and less than 50 reads; (ii) be outside of repeat sequences (based on the UCSC Genome Browser chicken repeat annotations); (iii) not correspond to a CpG prone site and (iv) be outside exons and untranslated regions (UTRs) that are likely to be affected by natural selection. In order to obtain data on synonymous and non-synonymous polymorphisms, we separately considered the corresponding positions within exons, still fulfilling criteria i) to iii).

Exons and UTRs coordinates were obtained through the BioMart query interface (http://www.ensembl.org/biomart/martview) [54]. When no UTR was annotated for a transcript, we excluded 77 bp upstream of the transcript (i.e. in 5' direction) and 372 bp downstream of the transcript (i.e. in 3' direction), sizes corresponding to the mean lengths of annotated 5' and 3' UTRs in chicken, respectively. A CpG prone site was defined as any C followed by a G or any G preceded by a C, as well as any C/T polymorphism followed by a G or any G/A polymorphism preceded by a C, following [55].

### SNP calling and estimates of diversity level

To be called a SNP, we followed the approach by Rubin et al. [25], that is we applied the criterion that the alternative nucleotide state, i.e. the non-reference allele, must be supported by at least three reads different to the nucleotide state found in the reference genome. While diversity estimates were obtained for RJF, broiler and layer populations separately, the support of the alternative nucleotide state was based on combined data of multiple populations. For example, consider a hypothetical position with a C in the reference genome. If the broiler population had two reads with A and one read with C at this position, and the layer population had one with A and two with C, the position was called a SNP in both layer and broiler populations, because the number of non-reference alleles (i.e., A) summed up to 3. Once the SNPs were called, we validated our SNP calls with those SNPs called by Rubin et al. [25], and only used consistently called SNPs.

After the validation step, we computed the number of SNPs per non-overlapping window (SNP density) for 1 Mb, 500 kb, 250 kb and 100 kb windows. Additionally, we computed the mean coverage per window as the average read depth per validated genomic position. These tasks were performed using in-house perl scripts. We determined the number of synonymous and non-synonymous SNPs per synonymous and non-synonymous sites by in-house C++ scripts incorporating the Bio++ library [56].

In order to correct SNP density estimates for variation in coverage, we estimated diversity level following an approach by Cutter and Moses [32]:

$$\hat{\theta }=\left[\mathrm{SNP}\right]/\log\left(n-1\right)$$

vwhere [*SNP*] represents the SNP density and *n* denotes the mean coverage. This computation was performed for all four window sizes for intergenic SNPs, where in the following *θ* is referred to as diversity level. For synonymous and non-synonymous polymorphisms the estimation was only performed for 1 Mb windows in order to ensure a reasonable signal-to-noise ratio. Here, the mean coverage was exclusively based on the read depth of synonymous and non-synonymous sites, respectively. Synonymous and non-synonymous diversity levels are in the following referred to as p_S_ and p_N_, respectively. Note that the absolute values of our diversity estimates are not directly comparable to other studies in chicken, because we used NGS data based on pooled samples rather than Sanger sequencing data and we employed stringent filtering criteria.

### Sequence data

Sequence alignments of orthologous intergenic regions for chicken, turkey (*Meleagris gallopavo*) and zebra finch (*Taeniopygia guttata*) were retrieved from whole-genome alignments from the Ensembl database release 61 via the Ensembl perl Application Programme Interfaces (APIs). We partitioned the whole-genome alignments into the four window sizes stated above, respectively, each with reference to the chicken genome. Then positions of transcribed regions including UTRs were established and masked with reference to the chicken genome. For each dataset, we restricted the data to windows with a minimum of 10,000 unambiguous sites, of which there were 1,038 windows of size 1 Mb, 2,040 of size 500 kb, 3,986 of size 250 kb and 9,205 of size 100 kb.

Coding sequence (CDS) alignments of orthologous genes in chicken, turkey and zebra finch were retrieved through the protein trees from the Ensembl database release 61 via Ensembl perl APIs. Orthologous genes were restricted to one-to-one orthologs, as defined in the Ensembl database. Alignments of one-to-one orthologs were then concatenated based on the windows defined for intergenic regions; for genes spanning more than one window, the different parts were assigned to the respective window. Windows containing no one-to-one orthologs were discarded for downstream analysis. Sequence alignments were cleaned for possible misaligned sites running Gblocks with default parameter settings [57].

### Estimation of divergence, d_N_, d_S_, gene density and recombination rate

We estimated chicken-specific divergence for intergenic regions as the branch length between chicken and its common ancestor with turkey after all sites showing a CpG in any of chicken, turkey and zebra finch had been masked from the 3-way alignments. Estimation of branch length was based on the PAML software package version 4.1 and the general time-reversible substitution model implemented in baseml. CpG sites were masked from the alignments in order to avoid substitution rate variation caused by hypermutability of CpG sites and thus divergence being affected by the local CpG content.

We estimated chicken-specific rates of non-synonymous (d_N_) and synonymous (d_S_) substitution for the concatenated CDS alignments using PAML software package version 4.1. CDS alignments were concatenated in a given window of size 1 Mb, 500 kb and 250 kb, respectively. To estimate chicken-specific d_S_ and d_N_, we then used the branch-model implemented in codeml allowing the d_N_/d_S_ ratio to vary between the chicken branch and the remaining tree.

We estimated gene density as the proportion of exonic sites within a particular window. We also included UTRs and exon-intron boundaries as “genic” sites, as they might represent functionally important sequences. For the exon-intron boundaries, we included 10 bp of intronic sequence after the end and before the start of each exon [58].

We computed the sex-averaged chicken recombination rate using data from Groenen et al. [36] and the WUGSC 2.1 chicken assembly. Recombination rate per 1 Mb window was computed as the mean recombination rate (genetic distance/physical distance) between markers weighted by the physical distance between markers, ranging from 0 – 28.6 cM per 1 Mb window (a histogram of recombination rate is provided in the Additional file 1: Figure S3).

### Statistical analysis

To investigate the degree of common variation in genetic diversity between the three chicken populations we performed PCA of local diversity level for 1 Mb, 500 kb and 250 kb non-overlapping windows. The computation of the PCs was performed via an eigenvalue-decomposition of the associated covariance matrix as implemented in the “princomp” function of the statistical software package R version 2.9.2. The degree of common variation was then defined as the leading PC, i.e. PC I, and as a local measure of the common genetic variation in the three populations we projected diversity levels on PC I, which can be seen as a smoothing function through genetic diversity in all three chicken populations.

We performed multi-linear regression analysis for diversity level grouped into four groups: common genetic variation in diversity level (PC I) and variation in each of the three chicken populations separately. For all four groups we conducted regression analysis based on 880 out of 1,038 non-overlapping windows of size 1 Mb, where data on the six possible explanatory variables recombination rate, divergence, gene density, GC content, d_S_ and d_N_ were available. We further conducted regression analysis for 1,623 windows of size 500 kb and 2,651 windows of size 250 kb. We transformed the explanatory variables in order to reduce the skewness in their distributions. Recombination rate was log-transformed to base 10, after adding a constant of 1 in order to allow for zero rate values. All the other explanatory variables were transformed by the square root. Regression analysis was then performed after Z-transformation of the explanatory variables, which means standardization of the mean value to 0 and of the standard deviation to 1.

We performed PLSR analysis, a regression setup that accounts for multi-collinearity in the explanatory variables [59]. As stated above for the multi-linear regression analysis, explanatory variables were first transformed to reduce the skewness in their distributions and then Z-transformed. In addition, also diversity level estimates were also Z-transformed. PLSR was then conducted for diversity level estimates based on 1 Mb windows for each of the four groups of genetic variation separately.

We performed an autocorrelation analysis of local diversity level and divergence based on 100 kb windows. This was done computing Pearson correlation coefficients and their *p*-values for measurements of nearest neighboring windows (k = 1) up to windows lying 5 Mb apart (k = 50).

Genomic regions of candidate selective sweeps identified by Rubin and colleagues were mapped onto the 1 Mb windows used throughout our analysis [25]. Averages of diversity levels, p_S_ and p_N_, as well as averages of the six explanatory variables used in the above described regression analysis were determined for the candidate loci as the arithmetic means of the respective 1 Mb windows. Genome-wide averages of the same variables were determined as the arithmetic means over all windows. To assess the significance in the difference between the averages for the candidate loci and the genome-wide averages we bootstrapped the genome-wide averages based on a sample size of 9999 and computed *p*-values based on their bootstrap confidence intervals.

All statistical analyses were performed with the software package R version 2.9.2.

## Abbreviations

RJF: Red jungle fowl;PCA: Principal component analysis;PC: Principal component;PLSR: Partial least square regression;Ne: Effective population size;dS: Synonymous divergence;dN: Non-synonymous divergence;pS: Synonymous diversity;pN: Non-synonymous diversity;gBGC: GC-biased gene conversion;API: Application Programme Interface;CDS: Coding sequence

## Competing interests

The authors declare that they have no competing interests.

## Authors’ contributions

BN and CFM performed the bioinformatics analysis. CFM performed the statistical analysis. CFM and HE conceived and designed the study. BN, CFM and HE drafted the manuscript and all authors approved the final manuscript.

## Supplementary Material

Additional file 1: Figure S1 Biplots of the first two principal components (PCs) for diversity level across the genome for three window sizes, 1 Mb, 500 kb and 250 kb. In each graph one black dot represents one window, where observations of diversity level in the three populations are projected into the space of the first two PCs. The red arrows labeled RJF, Broiler and Layer display the loadings for diversity level in the respective population. Arrows which represent the loadings of the three populations on PC I and PC II showed a strong component along PC I all pointing in the same direction and a much weaker along PC II. **Figure S2.** Amount of variation in local diversity level in each of the three chicken populations explained by the different explanatory variables based on PLSR analysis. **Figure S3.** Histogram of recombination rate in cM per 1 Mb window. **Table S1:** Summary of genome-wide averages and 95% bootstrap confidence intervals (CIs) of diversity level, p_S_, p_N_ and p_N_/p_S_ estimates for the three different chicken populations. For diversity level data for three different window sizes are included whereas for p_S_, p_N_ and p_N_/p_S_ only 1 Mb windows are considered due smaller sample sizes. **Table S2:** Estimates and p-values in a multi-linear regression analysis for six possible explanatory variables of chicken diversity level in 500 kb windows for the three populations and for common genetic variation. Estimates and p-values significant at a threshold < 0.001 are highlighted in bold. **Table S3:** Estimates and p-values in a multi-linear regression analysis for six possible explanatory variables of chicken diversity level in 250 kb windows for the three populations and for common genetic variation. Estimates and p-values significant at a threshold < 0.001 are highlighted in bold. **Table S4:** Estimates and p-values in multi-linear regression analysis for seven possible explanatory variables of chicken diversity levels in 1 Mb windows. Common variation reflects PC I of diversity level of all three populations. Estimates and p-values significant at a threshold < 0.001 are highlighted in bold. Click here for file
