# Supplementary material for: Genome-wide analysis in chicken reveals that local levels of genetic diversity are mainly governed by the rate of recombination
Source: BMC Genomics. 2013 Feb 8;14:86. doi: 10.1186/1471-2164-14-86 (PMC3600008; doi:10.1186/1471-2164-14-86)
Supplement: Additional file 1: Figure S1 — Biplots of the first two principal components (PCs) for diversity level across the genome for three window sizes, 1 Mb, 500 kb and 250 kb. In each graph one black dot represents one window, where observations of diversity level in the three populations are projected into the space of the first two PCs. The red arrows labeled RJF, Broiler and Layer display the loadings for diversity level in the respective population. Arrows which represent the loadings of the three populations on PC I and PC II showed a strong component along PC I all pointing in the same direction and a much weaker along PC II. Figure S2. Amount of variation in local diversity level in each of the three chicken populations explained by the different explanatory variables based on PLSR analysis. Figure S3. Histogram of recombination rate in cM per 1 Mb window. Table S1: Summary of genome-wide averages and 95% bootstrap confidence intervals (CIs) of diversity level, pS, pN and pN/pS estimates for the three different chicken populations. For diversity level data for three different window sizes are included whereas for pS, pN and pN/pS only 1 Mb windows are considered due smaller sample sizes. Table S2: Estimates and p-values in a multi-linear regression analysis for six possible explanatory variables of chicken diversity level in 500 kb windows for the three populations and for common genetic variation. Estimates and p-values significant at a threshold < 0.001 are highlighted in bold. Table S3: Estimates and p-values in a multi-linear regression analysis for six possible explanatory variables of chicken diversity level in 250 kb windows for the three populations and for common genetic variation. Estimates and p-values significant at a threshold < 0.001 are highlighted in bold. Table S4: Estimates and p-values in multi-linear regression analysis for seven possible explanatory variables of chicken diversity levels in 1 Mb windows. Common variation reflects PC I of diversity level of all three populatio [file 1471-2164-14-86-S1.pdf]

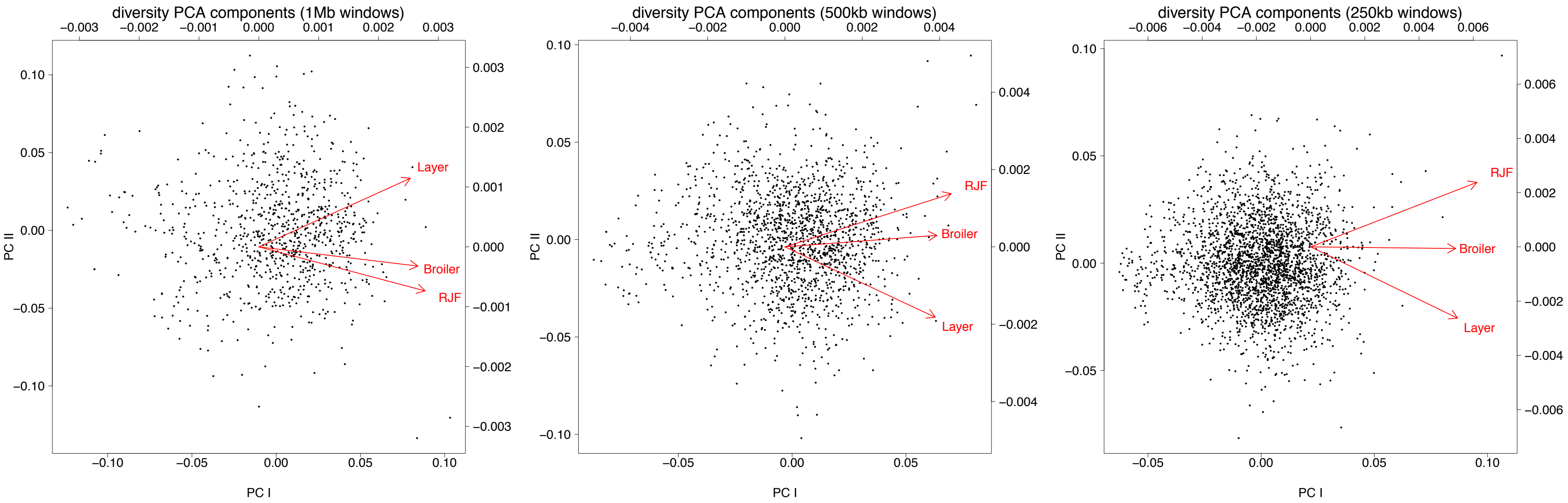

Figure S1. Biplots of the first two principal components (PCs) for diversity level across the genome for three window sizes, 1 Mb, 500 kb and 250 kb. In each graph one black dot represents one window, where observations of diversity level in the three populations are projected into the space of the first two PCs. The red arrows labeled RJF, Broiler and Layer display the loadings for diversity level in the respective population. Arrows which represent the loadings of the three populations on PC I and PC II showed a strong component along PC I all pointing in the same direction and a much weaker along PC II.

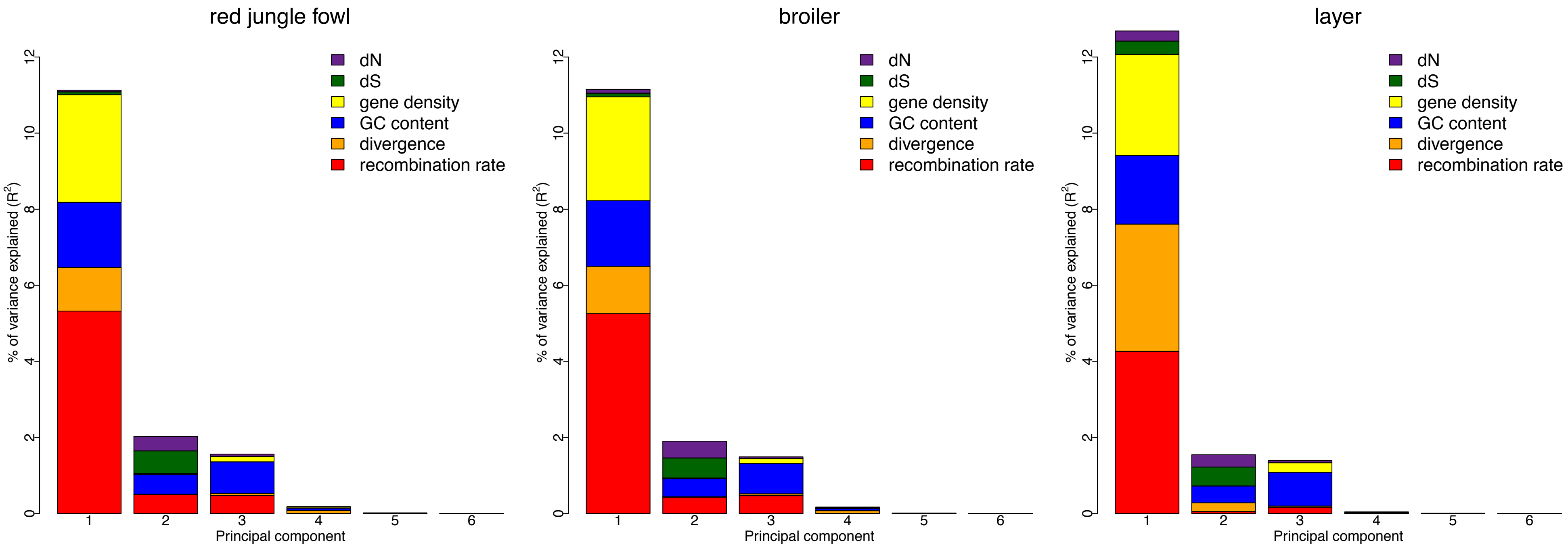

Figure S2. Amount of variation in local diversity level in each of the three chicken populations explained by the different explanatory variables based on PLSR analysis.

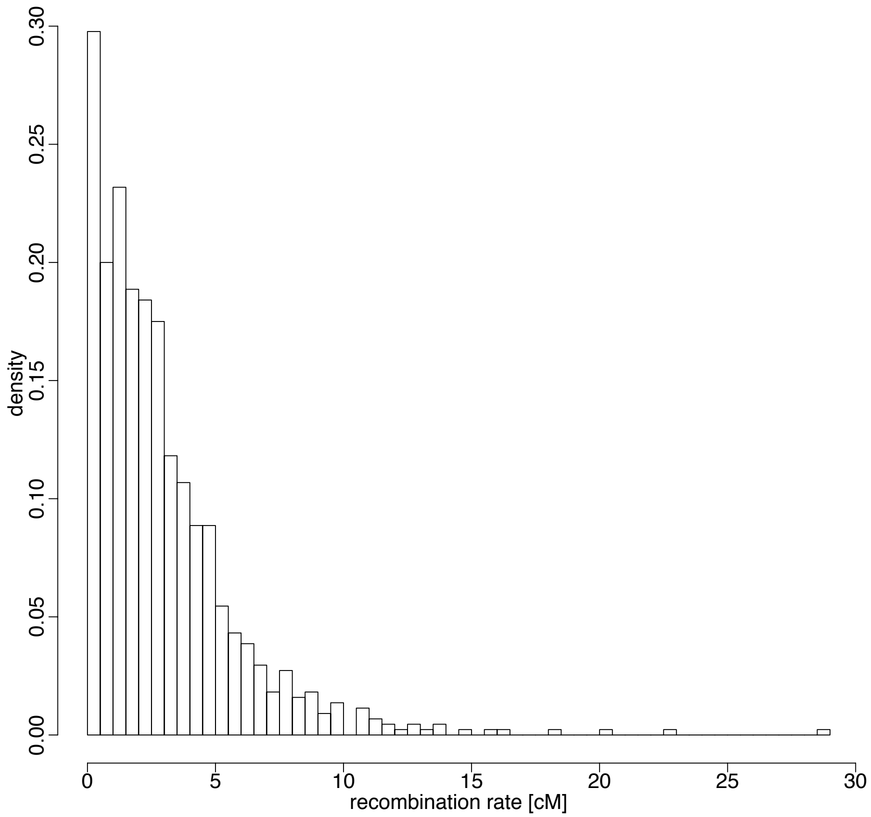

Figure S3. Histogram of recombination rate in cM per 1 Mb window.

Table S1: Summary of genome-wide averages and 95% bootstrap confidence intervals (CIs) of diversity level, p_S, p_N and p_N/p_S estimates for the three different chicken populations. For diversity level data for three different window sizes are included whereas for p_S, p_N and p_N/p_S only 1 Mb windows are considered due smaller sample sizes.

	window size	red jungle fowl		broiler		layer	
diversity × 10^4	1 Mb	3.69	(3.62, 3.75)	3.42	(3.36, 3.48)	3.06	(3.00, 3.12)
	500 kb	3.98	(3.92, 4.03)	3.61	(3.56, 3.66)	3.27	(3.22, 3.32)
	250 kb	4.07	(4.02, 4.11)	3.70	(3.66, 3.74)	3.36	(3.31, 3.40)
$p_S \times 10^4$	1 Mb	5.12	(4.79, 5.49)	5.11	(4.90, 5.32)	4.66	(4.43, 4.89)
$p_N \times 10^5$	1 Mb	5.50	(4.90, 6.09)	5.38	(5.03, 5.81)	5.01	(4.61, 5.43)
p_N/p_S	1 Mb	0.107	(0.093, 0.122)	0.105	(0.098, 0.114)	0.108	(0.098, 0.119)

Table S2: Estimates and *p*-values in a multi-linear regression analysis for six possible explanatory variables of chicken diversity level in 500 kb windows for the three populations and for common genetic variation. Estimates and *p*-values significant at a threshold < 0.001 are highlighted in bold.

	red jungle fowl		broiler		layer		common	
	estimate	*p*	estimate	*p*	estimate	*p*	estimate	*p*
recombination rate	**2.45e-05**	**2.76e-14**	**2.29e-05**	**2.22e-15**	**1.85e-05**	**6.75e-10**	**3.82e-05**	**5.24e-15**
divergence	-5.34e-06	6.93e-02	-4.01e-06	1.29e-01	**-1.31e-05**	**2.06e-06**	-1.28e-05	4.11e-02
gene density	**2.33e-05**	**3.52e-11**	**2.11e-05**	**2.73e-11**	**2.30e-05**	**3.25e-12**	**3.89e-05**	**3.52e-13**
GC content	**-1.53e-05**	**7.83e-05**	**-1.32e-05**	**1.38e-04**	-1.09e-05	2.63e-01	**-2.28e-05**	**9.83e-05**
d_S	-1.10e-06	7.79e-01	-3.75e-06	2.86e-01	-3.93e-06	2.84e01	-4.96e-06	4.03e-01
d_N	-3.09e-06	4.28e-01	-1.63e-06	6.41e-01	-4.01e-06	2.72e-01	-5.04e-06	3.94e-01
	Multiple $R^2 = 0.0736$		Multiple $R^2 = 0.0788$		Multiple $R^2 = 0.0818$		Multiple $R^2 = 0.0875$	
	$p < 2.2e-16$		$p < 2.2e-16$		$p < 2.2e-16$		$p < 2.2e-16$	

Table S3: Estimates and *p*-values in a multi-linear regression analysis for six possible explanatory variables of chicken diversity level in 250 kb windows for the three populations and for common genetic variation. Estimates and *p*-values significant at a threshold < 0.001 are highlighted in bold.

	red jungle fowl		broiler		layer		common	
	estimate	*p*	estimate	*p*	estimate	*p*	estimate	*p*
recombination rate	**2.02e-05**	**6.83e-14**	**1.74e-05**	**6.12e-14**	**1.377e-05**	**2.49e-08**	**2.98e-05**	**4.50e-14**
divergence	2.16e-06	4.05e-01	1.79e-06	4.21e-01	-7.288e-06	2.20e-03	-1.71e-06	6.53e-01
gene density	**2.28e-05**	**9.48e-15**	**2.04e-05**	**8.04e-16**	**2.246e-05**	**< 2e-16**	**3.79e-05**	**< 2e-16**
GC content	**-1.73e-05**	**2.44e-08**	**-1.53e-05**	**9.22e-09**	**-1.105e-05**	**1.01e-04**	**-2.53e-05**	**2.41e-08**
d_S	-6.69e-06	3.49e-02	-5.80e-06	3.34e-02	-5.728e-06	4.93e-02	-1.06e-05	2.33e-02
d_N	-4.68e-06	1.40e-01	-4.50e-06	9.92e-02	-5.388e-06	6.46e-02	-8.39e-06	7.15 e-02
	Multiple R^2 = 0.0497		Multiple R^2 = 0.0519		Multiple R^2 = 0.0456		Multiple R^2 = 0.0562	
	p < 2.2e-16		p < 2.2e-16		p < 2.2e-16		p < 2.2e-16	

Table S4: Estimates and *p*-values in multi-linear regression analysis for seven possible explanatory variables of chicken diversity levels in 1 Mb windows. Common variation reflects PC I of diversity level of all three populations. Estimates and *p*-values significant at a threshold < 0.001 are highlighted in bold.

	red jungle fowl		broiler		layer		common	
	estimate	*p*	estimate	*p*	estimate	*p*	estimate	*p*
recombination rate	**3.28e-05**	**3.37e-16**	**3.05e-05**	**8.39e-16**	**2.24e-05**	**9.78e-10**	**4.97e-05**	**1.38e-15**
divergence	-7.16e-06	3.36e-02	-6.87e-06	3.10e-02	**-1.42e-05**	**5.11e-06**	-1.61e-05	2.09e-03
gene density	**2.36e-05**	**2.32e-08**	**2.18e-05**	**4.64e-08**	**2.50e-05**	**1.53e-10**	**4.05e-05**	**6.54e-10**
GC content	-6.90e-06	9.74e-02	-6.80e-06	8.40e-02	-8.45e-06	2.77e-02	-1.27e-05	4.87e-02
d_S	-4.22e-06	3.03e-01	-5.30e-06	1.72e-01	-6.92e-06	6.73e-02	9.41e-06	1.39e-01
d_N	-1.40e-05	8.86e-03	-1.18e-05	1.95e-02	7.83e-07	8.74e-01	-1.49e-05	7.37e-02
chromosome size	5.16e-06	1.79e-01	5.95e-06	1.01e-01	**2.59e-05**	**5.63e-13**	**2.08e-05**	**5.05e-04**
	Multiple R^2 = 0.1531		Multiple R^2 = 0.1535		Multiple R^2 = 0.2087		Multiple R^2 = 0.1807	
	$p < 2.2e-16$		$p < 2.2e-16$		$p < 2.2e-16$		$p < 2.2e-16$	
